# The multifaceted care-seeking practices among caregivers of children with cerebral palsy: Perspectives from mothers and providers in Ghana

**DOI:** 10.1371/journal.pone.0258650

**Published:** 2021-10-27

**Authors:** Victoria Fonzi, Blessed Sheriff, Sarah Dalglish, Adote Anum, Emmanuella Dwomo Agyei, Devin Diggs, Loretta Eboigbe, Prince Gyebi Owusu, Kwame S. Sakyi

**Affiliations:** 1 Center for Learning and Childhood Development, Accra, Ghana; 2 College of Public Health, University of Georgia, Athens, Georgia, United States of America; 3 Warren Alpert Medical School, Brown University, Providence, Rhode Island, United States of America; 4 School of Public Health, Johns Hopkins University, Baltimore, Maryland, United States of America; 5 Department of Psychology, University of Ghana, Accra, Ghana; 6 Department of Public and Environmental Wellness, School of Health Sciences, Oakland University, Rochester, Michigan, United States of America; Waikato Institute of Technology, NEW ZEALAND

## Abstract

**Background:**

Research on cerebral palsy (CP) has lacked emphasis on knowledge and treatment practices among caregivers, particularly in low- and middle-income countries where socio-cultural contexts encourage a variety of treatment alternatives. In this study, we explored the beliefs and experiences that motivate care-seeking practices among caregivers of children with CP in Ghana.

**Methods:**

Semi-structured interviews were conducted with 25 caregivers, 10 medical providers, and 5 alternative providers in the Greater Accra Region. Participant interviews were analyzed using principles adapted from grounded theory. A conceptual model was constructed to illustrate salient patterns and motivational factors influencing care-seeking practices.

**Results:**

Participants’ experiences showed that caregivers initially sought physiotherapy and prescription medications from medical providers. Many of them then transitioned to alternative methods to search for a cure or address specific CP symptoms. Over time, most caregivers discontinued both medical and alternative care in favor of at-home treatment. A few withdrew completely from all forms of care. Cost of treatment, caregiver burden, and stigma strongly inhibited care-seeking outside the home.

**Conclusion:**

Although caregivers were open to exploring a variety of treatment options, at-home treatment was preferred by long-time caregivers for its convenience, low cost, and adaptability to patient and caregiver needs.

## Introduction

Cerebral palsy (CP) is a neurodevelopmental disability that begins in infancy and persists throughout an individual’s lifespan. It is the most common physical disability in childhood, affecting 17 million children worldwide [1]. The average worldwide incidence of CP is 1 in 500 births [2], but in Ghana cases of cerebral palsy are particularly high, affecting 1 in every 300 newborns [3]. In low-resource settings, the burden of this disability is further aggravated by the severity of its associated symptoms and common comorbidities–particularly epilepsy and acute cognitive and motor impairments [4,5]. Studies in Ghana show that children with CP commonly suffer from a slew of other comorbidities, especially malnutrition, delayed growth, and aspiration difficulties [3,5]. The range of co-occurring conditions and indications associated with cerebral palsy in Ghana and other low-resource contexts highlights the importance of prompt and effective care-seeking to both prevent and manage complications.

A number of treatments for children with CP are supported by significant clinical evidence. In a recent systematic review, researchers analyzed 64 discrete interventions for cerebral palsy (including both orthodox and alternative methods) with the aim of describing their effectiveness and providing recommendations for standard of care. Of the eight interventions supported by high quality evidence, six were medical interventions or interventions that required some form of clinical training to administer [6]. The majority of these interventions involved prescription medications for motor impairment, as well as functional training/therapy [6]. In countries with lower cerebral palsy mortality rates, multidisciplinary medical care is delivered by specialized health professionals such as speech therapists, occupational therapists, physiotherapists, and dietitians [7,8].

The use of complementary and/or alternative methods is the norm in care-seeking among sub-Saharan Africans, 80% of whom use some form of alternative treatment (mainly herbalism and spiritual healing) in their health care, for all types of diseases and conditions [9]. The Ghana Traditional and Alternative Medicine Directorate registers traditional practitioners and offers safety and efficacy testing for traditional medicines [10]. The use of alternative therapeutics in Ghana is well-documented in the case of cerebral palsy and other developmental disabilities due to prevalent cultural beliefs that the cause of such disabilities is likely spiritual [11,12]. However, the benefits of many alternative and complementary care methods for the treatment of cerebral palsy are unclear at best, mainly due to insufficient evidence or poorly designed efficacy studies, and most alternative medicines have yet to be tested for safety [10,13,14]. There are also many anecdotal reports that the rituals performed in traditional medicine for the treatment of cerebral palsy in Ghana have been ineffective or dangerous, oftentimes amounting to infanticide [11,15].

The existence of diverse care methods for cerebral palsy has been explored in the research literature, and the experience of caregivers of children with cerebral palsy has been thoroughly documented in high-income settings. The research on cerebral palsy in Africa is very limited. There are significant gaps in knowledge on CP causes, risk factors, and prevalence. In Ghana, research on children with special needs and their caregivers hardly focuses on treatment and treatment practices. Most research focuses on caregiver burden [4], caregiver experiences [5,11,16], or coping [17]. This existing focus creates a gap in research on treatment practices among the caregivers who are integral in the care of CP, whether in medical, alternative, or home settings. Thus, policymakers, physicians, and stakeholders have a poor evidence base for understanding and improving care-seeking. Using a qualitative approach, this study examines the multifaceted care-seeking behaviors of mothers of children with cerebral palsy in Ghana, exploring beliefs, expectations, and experiences which motivate different care-seeking methods, as well as withdrawal from care.

## Methods

This qualitative study investigated care-seeking strategies to treat children with cerebral palsy in various clinical, alternative, and home-based settings. The epistemological underpinning of the study was constructivism, and the methodological underpinning was grounded theory [18,19]. As such, no pre-existing theoretical framework was used to explain transitions through medical, alternative, and home-based care systems. Instead, the conceptual framework created was grounded in the data collected through an inductive coding approach [19]. Our methodological approach deviated from traditional grounded theory in that the literature review was not reserved until the end of data collection [18] (a common modification made by qualitative researchers). We used the consolidated criteria for reporting qualitative research (COREQ) guidelines to guide the reporting of the study [20].

### Setting

This study was conducted in the Greater Accra Region of Ghana, the country’s most densely populated region. Princess Marie Louise Children’s Hospital is a government health facility that serves as the region’s premier pediatric health center. All healthcare worker participants in this study are employed by Princess Marie Louise Children’s Hospital, and most caregiver participants report having sought treatment at this hospital. Princess Marie Louise has established a medical standard of care for pediatric CP through a combination of neurodevelopmental, physiotherapy, and nutrition clinics. In the neurodevelopmental clinic, pediatric physicians diagnose developmental disabilities and coordinate care regimens. The physiotherapy clinic performs functional training with CP patients three days a week to improve motor skills and spasticity. Physiotherapists work in conjunction with pediatricians to improve drug dosage for medications which impact muscle tone. In the nutrition clinic, an on-site nutritionist provides specialized nutritional counseling to caregivers. Caregivers also frequently receive advice and emotional support from peers through virtual groups that are not affiliated with the hospital.

### Participants, recruitment, and sampling

Three groups of participants were recruited for this study: (1) mothers of children with cerebral palsy, (2) medical providers, and (3) alternative care providers. Inclusion and exclusion criteria are listed in Table 1. Recruitment occurred simultaneously with analysis and continued until saturation was achieved on study questions (i.e., new interviews did not yield new information on the study questions). The recruitment and sampling of each type of participant is described in turn below.

**Table 1:** Inclusion/Exclusion criteria for participants.

Participants	Inclusion Criteria	Exclusion Criteria
Mothers of children with cerebral palsy	• Female, age 18–49 • Currently residing within Ghana • Has one or more children (aged 0–18 years) with CP	• Below 18 years of age or above 49 years of age • Not currently residing within Ghana • Not the mother of a child with CP • Has a child with CP who is over the age of 18 • No consent given
Health workers–pediatricians, physiotherapists, occupational therapists, social workers	• Currently employed by Princess Marie Louise Children’s Hospital as a health worker • Some experience treating children with CP	• Not currently employed by Princess Marie Louise Hospital • No experience treating children with CP • No consent given
Alternative/complementary medicine providers–religious leaders, herbalists, traditional healers	• Some experience treating children with CP as a non-medical provider • Currently practicing in Accra	• No experience treating children with CP • Not currently practicing in Accra • No consent given

#### Mothers

Unpublished preliminary studies conducted by the Center for Learning and Childhood Development, the local partner for this study, demonstrated that the primary caregivers of children with cerebral palsy in Ghana are mothers. Mothers were initially recruited by phone from a large support group for mothers of children with cerebral palsy in Ghana on its WhatsApp platform. This group serves families from diverse socioeconomic backgrounds. Ten mothers were randomly selected and screened for inclusion and exclusion criteria. Each mother who met the inclusion criteria was called and asked to participate in a face-to-face interview at a location of her choice, often at or near her home. Participants were also asked to refer or provide contact information for other mothers of children with cerebral palsy who may be interested in the study. This snowball sampling technique was used to recruit the remaining mothers in the study. Participant selection occurred concurrently with emerging theme analysis.

#### Medical care providers

Medical providers were purposively sampled from the neurodevelopmental, physiotherapy, and nutrition clinics and the emergency department at Princess Marie Louise Children’s Hospital, which attends to a high traffic of cerebral palsy patients. All medical care provider participants were recruited and interviewed in-person at the hospital. Researchers screened potential participants for inclusion and exclusion criteria, and all eligible participants were interviewed.

#### Alternative care providers

Two sampling methods were used to recruit alternative care providers. First, a snowball sampling technique was employed, by which mothers were asked to provide phone numbers of alternative care providers they had used. Second, alternative care providers were purposively sampled from a list provided by the Ghana Mental Health Authority. All providers were called and screened for inclusion/exclusion criteria by researchers. Consenting participants who met inclusion criteria were interviewed at their places of work.

### Data collection

Each participant completed a background questionnaire with demographic data, including marital status, educational level, occupation, age, and household income. Participants were then asked to participate in a freelisting exercise [21] in which they listed all methods they personally used or prescribed in the treatment of cerebral palsy. Finally, an in-depth face-to-face interview lasting approximately 20–50 minutes was conducted.

Authors VF, BS, EDA, DD, and LE, undergraduate university students at the time of data collection, served as interviewers. They were supervised by authors KS (a qualitative social scientist with a PhD in public health), and PO (a developmental disability expert and qualitative researcher in Ghana). Both KS and PO had established relationships with the Princess Marie Louise Hospital and the mother support group, from which participants were selected. The four female and one male interviewer received a week of intensive training in conducting interviews using semi-structured interview guides with pre-tested, open-ended questions. Interview guides used for the provider and caregiver interviews are provided as a supplement (S1 and S2 Tables, respectively). Interviewers were trained to follow up on participant responses in a neutral tone and take note of body language, tone, and the environmental context of each interview in extensive field notes following the interview [22]. With participant consent, interviews were conducted in a private space with only the participant and researcher present. General topics included: (1) social perceptions of cerebral palsy, (2) outcome expectations surrounding different forms of care, (3) challenges with seeking medical care, (4) lifestyle and health management of children with CP, and (5) knowledge about available medical/alternative health care resources for children with CP. None of the eligible participants refused to participate in the study.

### Analysis

Interviews were transcribed and translated from Twi, the predominant local language, into English when necessary. One interview was not recorded at the request of the participant and thus was reconstructed using extensive field notes. Interview analysis adapted grounded theory analytical techniques of axial coding, line-by-line coding, memoing, constant comparative analysis, and conceptual framework development [19]. We modified the grounded theory analysis by counting the number of participants who made transitions from the different medical systems with the purpose of understanding how salient each category was within the data. Semi-quantification, such as frequency analysis, in qualitative studies can enable researchers to recognize patterns across data, improve transparency of analysis, and enhance precision of statements of findings [23].

In the first phase of analysis, four interviews were triple-coded inductively for recurring concepts, ideas, and storylines. An initial codebook was constructed based on categories of treatment methods and care-seeking rationale. The remaining interviews were conducted to ensure each core category was saturated, and the resulting transcripts were coded simultaneously with ongoing data collection. Regular research team meetings were held to compare and consolidate core thematic categories. Coding was conducted by all five interviewers using Dedoose version 8.2.14 [24].

### Ethics

Only adults who provided written consent were permitted to participate in this study. To avoid attaching participant names to any written or recorded data, data was labeled using randomly assigned participant identification numbers. Recorded data was transcribed by the interviewer and shared internally with the research team in a written, non-identifiable form. Deidentified background questionnaires and full transcribed interviews were only shared among members of the research team. All potentially identifying information were excluded from the final results.

Participants were compensated with 20 Ghanaian Cedis (approximately US $3.46). The protocol for this study was approved by the Ghana Health Services Ethics Review Board (Protocol ID: GHS-ERC 025/04/18).

## Results

**Fig 1:**
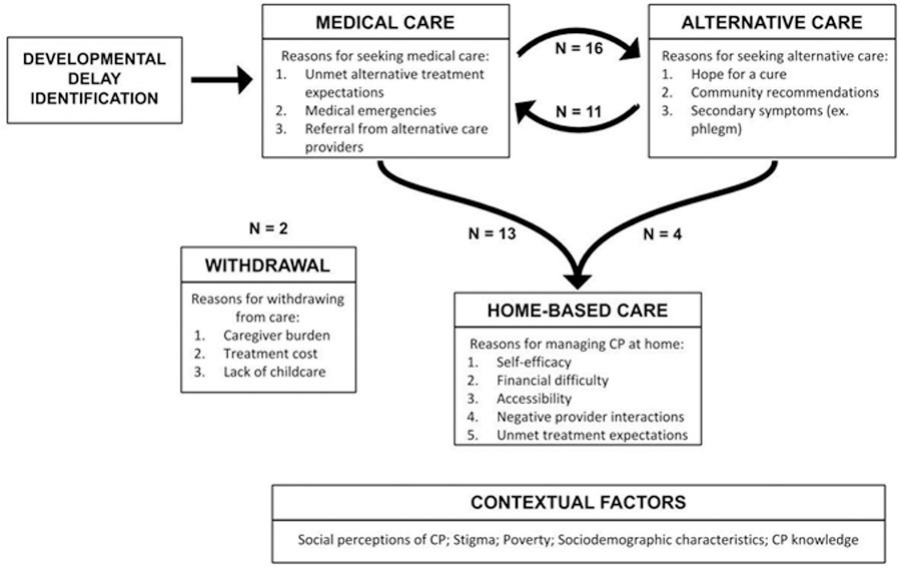
Key care-seeking factors for children with cerebral palsy (CP), N = 25. Common care-seeking transitions with rationales as reported by caregivers and providers. N values reflect the number of caregiver participants who made each transition and are not generalizable beyond this study sample.

Demographic characteristics of participants are given in Table 2 (caregivers) and Table 3 (providers). All caregivers (N = 25) received a cerebral palsy diagnosis within the medical care system. From there, caregivers sought treatment from medical providers, alternative providers, or both. Over half of caregivers (N = 16) reported disengaging from medical and/or alternative care over time, shifting to providing CP treatments at home. A small subset of caregivers (N = 3) withdrew from treating their children’s CP altogether and only addressed their children’s daily needs (eating, bathing, dressing, toileting, etc.). Major reasons for transitioning between medical care, alternative care, and home-based care strategies are outlined in Fig 1. One caregiver’s representative experience is provided (S1 File).

**Table 2:** Background characteristics of caregivers, N = 25.

Characteristics	Total N (%)
Categorical variables	
Education level	
Below secondary school	6 (24)
Completed secondary school	7 (28)
Some college, no degree*	1 (4)
College degree	7 (28)
Master’s degree	4 (16)
Marital status	
Married	16 (64)
Single	5 (20)
Separated	2 (8)
Divorced	2 (8)
Employment status	
Employed	13 (52)
Unemployed	12 (48)
Monthly household income	
< 100 GHC	3 (12)
101–450 GHC	3 (12)
451–1000 GHC	8 (32)
> 1000 GHC	9 (36)
Do not know/No response	2 (8)
Continuous variables	Mean (range)
Age	37.2 (29–46)

“Some college, no degree” includes any participants who finished secondary school and took one or more university courses but did not receive a degree.

**Table 3:** Background characteristics of providers, N = 15.

Characteristics	Total N (%)	Medical provider, N (%)	Alternative provider, N (%)
Categorical variables			
Gender			
Male	4 (27)	0 (0)	4 (80)
Female	11 (73)	10 (100)	1 (20)
Type of Provider			
Pediatrician	2 (13)	2 (20)	0 (0)
Nurse	4 (27)	4 (40)	0 (0)
Physiotherapist	2 (13)	2 (20)	0 (0)
Physiotherapist assistant	1 (7)	1 (10)	0 (0)
Nutritionist	1 (7)	1 (10)	0 (0)
Religious healer	5 (33)	0 (0)	5 (100)
Length of occupation			
1–5 years	3 (20)	2 (20)	1 (20)
5–10 years	6 (40)	5 (50)	1 (20)
> 10 years	6 (40)	3 (30)	3 (60)
Continuous variables	Mean (range)		
Age (yrs)	38.4 (27–59)	34.2 (27–46)	47.8 (34–59)

### Care techniques for cerebral palsy

Medical treatments for CP primarily consisted of physiotherapy and prescription muscle relaxants such as Baclofen. Children with co-occurring epilepsy were additionally prescribed anti-seizure medications such as Clonazepam. Malnutrition, another commonly cited co-occurring condition, was treated with therapeutic food and nutritional coaching for parents.

Alternative treatments for CP depended heavily on the provider and often included a combination of herbal treatments, prayers, and spiritual counseling for caregivers. However, providers were reluctant to describe their methods and the types of herbs they utilized in specific detail.

Home care most commonly consisted of at-home physiotherapy, using items found around caregivers’ homes to simulate the assistive devices found at a physiotherapy clinic. A few mothers also managed co-occurring conditions with over-the-counter medication or homemade herbal concoctions rather than prescription medications.

### Factors influencing transition from medical care to alternative care

After initially seeking medical care, approximately two-thirds of caregivers turned to alternative providers, either for the entirety of their children’s treatment or for specific symptoms that were not addressed satisfactorily within the medical care system. Approximately half of those who turned to alternative care believed that these treatments were effective, while the other half saw no improvement in their children’s conditions.

For those who switched completely from medical to alternative care, primary motivators included a desire to exhaust all potential remedies and advice from fellow community members, most commonly family members. Most caregivers who reported seeking an alternative care provider said their decision was a last resort, often using the word “desperation” [for a cure]. After learning that the hospital could not provide a cure for CP, caregivers sought out alternative providers who promised a “quick fix,” often due to a misperception of CP as an acute condition rather than a chronic one, leading caregivers to become impatient with the slow pace of medical care.

So I was still desperate. I thought… it was just a mere sickness or illness. Thinking that they [alternative providers] would see something—the source of it–and then, we would resolve it. … so that is why I was going to them. Because I thought it was a mere sickness and it can be solved. It can be cured. [Mother, age 32]

They expect the process to be a done deal. ‘I come to the hospital. Within a week or two, my child should be okay.’ But it’s not like that. So they start seeking help elsewhere, doing other traditional medicines and churches and all of that. [Pediatric nurse]

Over a third of the caregivers who pursued alternative care did so in conjunction with medical care and only for specific secondary symptoms of CP that were not effectively controlled by medical treatment. Phlegm was by far the most cited symptom treated with alternative care methods, which involved inducing the child to regurgitate excessive mucus. Many caregivers said these treatments made it easier for the child to breathe.

She would drink it [herbal medication] and vomit the phlegms. [Mother, age 35]

Yeah, she really snored, but after [removing] the phlegms, it was okay. She hardly snores like that again. [Mother, age 45]

### Factors influencing transition from alternative care to medical care

Approximately three-fourths of the participants who sought alternative care eventually transitioned away from alternative treatments and back to the medical care system. Regardless of the reason for this initial alternative care-seeking, caregivers overwhelmingly cited unmet treatment expectations as their primary reason for discontinuing alternative care. The most common unmet expectation was a perceived lack of efficacy of alternative treatments:

Traditionalist. I took him to that place too. [I took him to] just the herbalist. He gave him some medicine. But now we stopped [going to the herbalist]… I didn’t see anything, so I stopped. [Mother, age 30]

[The caregivers] go to the herbalist, they go to [the] spiritualists and all that. So by the time they see that, ‘Oh, all these avenues are really not helping me… [and] my child is growing bigger and bigger with no activity, with no function,’ that’s when they now decide to come to the hospital. [Physiotherapist]

Even when herbal treatments were perceived to be effective, some caregivers were dissatisfied by the side effects. Although not mentioned by mothers, health providers also warned about potential complications of herbal treatments, including liver problems, heart failure, lung infection, bone infection, and kidney problems. Herbal medications’ side effects prompted caregivers to return to medical care, especially to treat medical emergencies:

Recently we were in the hospital. A child came, and he came with a liver problem, and during the interview, we realized that they had been giving him these [herbal] concoctions… You see, most of the time when they do the orthodox [alternative medicine] and then it gets to the extreme, then they bring the child to the hospital eventually. [Mother, age 35]

And sometimes, we even get them [children with CP] in the hospitals because they have had an infection as a result of the [herbal] concoctions they have been taking. [Physiotherapist]

Though not frequently mentioned by caregivers, alternative providers said they referred caregivers to hospitals when they determined that the child’s condition was physical, not spiritual, in nature. Notably, a few religious leaders reported repeatedly collaborating with health workers to provide care to CP children.

When we realize that the thing [CP] is not mainly … spiritual sickness, we let you see the doctor. [Religious leader]

When we started, they [children with CP] used to come here [the church]. About four nurses came here to take care of them. If the person can’t go there [the hospital], I call them. Even drips [IVs] they will come and do for me. [Bishop]

### Factors influencing transition from medical care to home-based care

After seeking CP treatment within medical settings, over half of caregivers ultimately transitioned to providing treatment primarily at home. Most mothers were drawn to home-based management of CP for four major reasons.

First, the majority of caregivers believed that they could treat their children’s conditions effectively on their own and even more efficiently than going to the hospital. This belief was especially common in regard to physiotherapy.

You know, I went to physio for two years, so now I can do it. I learned because when you go, they ask you to do it for him when at home. So I used to do it [go to physiotherapy] but now it is just me … I wake up early and massage his legs and hands. [Mother, age 36]

By the time we get home, she’s more sick, or it makes her spend more time at the hospital because the system doesn’t allow her to rest. Meanwhile, at home, I can allow her to sleep for as long as she wants. She wakes up, I grind the herbs, give an enema, and she’s fine. [Mother, age 38]

Secondly, half of the caregivers who transitioned to home-based management cited financial difficulties as a driving factor. Transportation costs were a significant contributor to this financial burden.

Yeah, I do it at home because… it got a bit expensive … [I]t was like I was wasting my money going there and then doing it myself. So I just stopped [going to physiotherapy]. [Mother, age 37]

A third reason had to do with physical access to medical treatment facilities, including the need for caregivers to physically carry children to appointments, which limited the distance they were able to travel. Relatedly, the difficulty of traveling long distances and waiting in lines made medical appointments less accessible:

I get tired before I get to the place [hospital]. These type[s] of children are heavy. [Mother, age 36].

So basically, I would commute to Accra via a bus for… four hours to get me here, to join a long queue at our teaching hospital. I naturally… felt it wasn’t worth it… When we got to three [years old], I had stopped all therapies, as in going to the hospitals, but I was doing it at home. [Mother, age not provided]

Lastly, a few caregivers who were able to manage the distance and financial costs of care cited negative experiences with healthcare providers as deterrents to seeking medical care. A few mothers were directly told by providers that medical care would be unhelpful. Others also said that healthcare workers’ negative attitudes and work ethic influenced their decision to leave medical care:

That was when I stopped going to Korle Bu [name of the tertiary hospital] because the doctors told me that there was nothing that they could do. [Mother, age 36]

Sometimes you go and what maybe you are expecting them [healthcare workers] to do to the child they will not do it because they complain they are tired and there are many [patients]. [Mother, age 32]

### Factors influencing transition from alternative care to home-based care

Approximately half of caregivers who pursued alternative treatments eventually transitioned to home-based care for their children. With the exception of one mother who made this transition from an exclusively alternative treatment regimen, caregivers who transitioned to home care generally shifted away from a combination of alternative and medical treatments.

The unmet treatment expectations discussed above served as a major cause for the transition from alternative to home-based care. These unmet expectations came in the form of side effects or a lack of improvement in the child’s condition:

I tried it [herbal medicine] one time, and I tried it for like a month. And it wasn’t even working with the [herbal] medicines, so I stopped. I still do [at-home] therapy with her every day. I’ve made it her lifestyle. I don’t do it for a set time, maybe 30 minutes of… pushing the wall, or when she’s watching TV, I ensure she sits on the floor and crosses her legs. [Mother, age 46]

The inefficacy of many alternative treatments led some mothers to perceive alternative care as a financial drain, making free home-based care a particularly appealing alternative:

I don’t know any pastor that has come on TV that I’ve never been to. I’ve been to all of them. So when I see a mother, I tell you, ‘Look, it won’t help you because me–I’ve gone to all of them. If there’s help there, I would have had it. So don’t go and waste your money.’ [Mother, age 37]

Despite being generally less expensive than medical care, alternative care was perceived to be financially exploitative:

For the pastors, some of them are liars. They will deceive you and get all your money. They can’t do anything for you. [Mother, age 29]

Many [mothers] have gone to fetish priests, many have been to these crock doctors and even those so-called men of God. And I will use this word wisely, they’ve been ripped off. [Religious leader]

In addition, some caregivers considered spiritual counseling to be actively harmful as providers frequently attributed blame for the child’s CP to family members. These negative interactions with alternative providers caused familial and emotional strain, pushing caregivers away from alternative care:

One tells you it is this person, the other tells you it is this person [that caused the CP]. So I realized that no, I have to stop going to these places [spiritual counselors]. If not, they will bring conflict between my family and even everyone around me. Because I will begin to see so-and-so as the cause of my problem. [Mother, age 32]

### Factors influencing withdrawal from all types of care

Few caregivers proceeded to completely or partially withdraw from CP symptom management. However, for those who did, the most common cause was caregiver burden, part of which was a negative emotional reaction to their child’s CP condition. When discussing their reasons for treatment withdrawal, caregivers often used the words “frustrated” and “stressed” to describe their experience in not seeing any improvements following treatment:

So later I got so frustrated, I locked myself indoors. I shut the door to the world. It was just myself and my daughter. And that is a mistake I regret. Maybe if I had not done so, maybe I would have gotten better [treatment] results than I have today. [Mother, age 33]

Treatment cost was also identified by caregivers as a reason for treatment withdrawal. Caregivers mentioned that treatment was too expensive or that they did not have money:

I don’t do anything [to treat the CP]. The price that she [the physiotherapist] mentioned was expensive: every month 1.5 million [150 Cedis] and then food. So now I don’t do anything. He is at home. [Mother, age 36]

Although less frequently cited, an unfilled need for childcare support was also identified as a contributor for treatment withdrawal. Caregivers’ other domestic and caregiving responsibilities were a major contributor to this need. As a result, they could not continue seeking treatment for their children:

My mother can’t see [she is blind], and I am the last born, so I have to take care of my mother and my child. I thought I was going to seek treatment at Nsawam, but I don’t have anybody to take care of my mother. That is why I didn’t go. [Mother, age 36]

### Contextual factors

Several key factors influenced all forms of care-seeking. Social perceptions of CP and resulting stigma were mentioned frequently in interviews with caregivers. There remains a widespread belief within Ghana that CP originates from spiritual, rather than physical, causes. Children with CP are sometimes seen as a family curse:

In Ghana, people believe it’s spiritual. Sometimes they even believe it’s a sin a forefather like a great-grandfather has done, and you are suffering for it. That is why you got a child like that. And the stigmatization in the society, it is serious. [Mother, age 35]

Others view children with CP as “river” or “snake” spirits and advocate for “escorting” the children back to the river or forest, a practice that amounts to infanticide:

[N]ormally they say that these children are river goddess children. You understand? That means they are spirits. They are not human beings… They come to disturb you. They come to do awful things. They [others in the community] will say ‘k) gya ne kwan’: ‘Go and escort the child.’ And it means go and kill the child. [Mother, age 37]

As a result of these social perceptions, nearly all caregivers reported experiencing stigma when taking their children outside the home for treatment, often discouraging care-seeking:

[P]eople stare at us. Yes. They stare too much and they ask unnecessary questions… Like I was carrying an alien or something. The stigma is just too bad. [Mother, age 36]

Stigma from family members and schools combined with heightened childcare needs forced most of the study participants to give up employment and stay home full-time with their children. As such, poverty also acted as a serious deterrent to all types of care-seeking, but particularly medical care, which was worsened when the stigma of having a child with CP caused many of the mothers to become single, creating new financial vulnerabilities:

Stigmatization is a barrier to therapy. Then, finances. Their spouses are not helping. Families are not helping with the care of the children and all that. Once they [caregivers] have a child with cerebral palsy, a lot of marriages have collapsed… so a lot of the husbands are nowhere to be found. So it’s also a barrier. [Physiotherapist]

At the same time, healthcare expenses represent a significant expenditure, further exacerbating often poor financial situations:

I thought since he is a sick, disabled child, everything is going to be free, but they take money wherever I go. When I enter this room they will take money, when I go here [the hospital] they will take money, but the [financial] situation is not good for me… I will use all my money to buy drugs, so that doesn’t allow me to follow the [orders of the] doctors who take care of him. [Mother, age 42]

Socio-demographic factors strongly influenced the type and extent of care sought. While stigma and poverty pushed some caregivers away from care-seeking outside the home, caregivers with higher education and socio-economic status appeared to feel comfortable independently researching and directing treatments for CP:

We [the mothers] discuss it because we know about them [the children] more than even the doctors… We Google a lot. So we go to him [the doctor] and say, ‘Ah, there’s this medication—it doesn’t have any effects on the child.’ [Mother, age 37]

This increased knowledge of CP promoted medical care-seeking and home-based management over alternative treatments.

## Discussion

This study is the first to examine the care-seeking behaviors of caregivers of children with CP in Ghana. After initial assessment and diagnosis, we found that most caregivers sought medical care, although many transitioned to alternative care in search of a cure or a treatment for secondary symptoms associated with cerebral palsy. Caregivers who gained understanding or knowledge of cerebral palsy or were dissatisfied with the results of alternative care usually returned to medical care. In the end, the majority of caregivers transitioned from both types of care to home-based management because it can be easily implemented and has a lesser financial drain on caregivers. A few caregivers either partially or completely withdrew from any form of care. The results further showed that stigma, poverty, and social perceptions of cerebral palsy influence the overall care-seeking behavior of caregivers of children with CP.

Transitions between types of care were motivated by both pull factors (benefits to treatment) and push factors (barriers to treatment). Studies of developmental disorders have cited a holistic approach [25,26], perceived efficacy [13,25], and personal preference [13,26] as key pull factors for alternative care use in Ghana. However, the strong community influence, desire to find answers, stigma, and need for symptom-specific treatment found in our study have not been widely discussed as motivating factors for general alternative care use. This discrepancy suggests a unique rationale for alternative care-seeking among mothers of children with cerebral palsy due to external factors and a lack of information rather than personal beliefs and preferences. These motivations for alternative care-seeking underscore the need for healthcare workers to inform caregivers adequately about potential risks and benefits of alternative treatments soon after diagnosis [27].

The results of this study add to the current literature by drawing attention to how a moral model of disability [28–30] and structural factors perpetuate unequal suffering and withdrawal from healthcare. Societies with a moral model of disability view disability as either a gift or a curse. Similar to views from other sub-Saharan African countries, Ghanaian society widely views children with cerebral palsy as family curses, spirits, or “river children” [31,32]. Our study found that one manifestation of this view, rooted in stigma, is divorce. As a social institution, marriage provides access to financial, emotional, informational, and instrumental resources. Participants in the study noted that divorce, which disadvantages women socially and economically in this setting [33], contributes to caregiver burden—physical, psychological, emotional, and financial burden. Caregiver burden is a proximate determinant of participants’ withdrawal from all forms of care, and financial difficulties directly resulted in choosing home-based care. Carona *et al*. have indicated that increased caregiver burden and overload in parents of children with neurodevelopmental conditions are positively correlated with behavioral disengagement, and both were negatively correlated with parents’ and their children’s quality of life [34].

Some caregivers choose to engage in home-based care, pursuing little or no medical and/or alternative services. However, home-based care in Ghana may be inadequate because most caregivers are single mothers with a severe lack of social support and education and poor access to the appropriate resources and tools to care for their children at home [12,35]. Due to the complicated nature of the disorder and its resulting sequelae, a high level of monitoring and consistent caregiving is required, which has proven impractical for these caregivers to manage alone, especially if they have added responsibilities of work and/or caring for other children [36]. As a consequence, the health of children with CP can suffer and the burden on caregivers can be untenable.

Relatedly, caregiver burden is maximized because Ghana’s National Health Insurance Scheme does not cover the costs of rehabilitation services for children living with a disability [37]. Some mothers in the study were surprised that they had to pay for all of the rehabilitation services their children needed. Moreover, the limited availability of medical and alternative care for children with disabilities also increased both physical (e.g. traveling long distances, carrying children on their backs) and financial costs (including transportation costs) of staying in medical care, all elements of caregiver burden. These systemic health system barriers, compounded by the socio-cultural factors, create unequal access to medical services, perpetuate vulnerability, and result in an unequal distribution of rehabilitation outcomes. Similar to other studies, some caregivers in this study felt that their children’s physical functioning worsened because they had stopped receiving medical care due to caregiver burden and poverty [11]. Future studies should examine how marital status, health insurance, and distance from rehabilitation services may combine to create disparities in rehabilitation outcomes and retention in care among children living with CP.

This study has a few limitations and some strengths. It was conducted in an urban setting; thus, the findings may not be transferable to rural areas. Also, not all provider perspectives are represented because medical provider participants were recruited from one public hospital, and alternative provider participants were all associated with religious organizations. Therefore, the opinions of private sector medical providers and non-religiously affiliated alternative providers such as herbalists are not described. In addition, most of the caregivers interviewed are a part of a cerebral palsy mothers’ support group, further limiting the diversity of participants. It is possible that mothers who are not part of this support group may have different experiences that are not captured. For example, most caregiver participants fell into a high-income group, potentially granting greater financial ability to explore a variety of CP treatments than a lower income group. Data on children’s ages is not available, so conclusions about how the length of time following a CP diagnosis affects treatment decisions cannot be drawn. Finally, the qualitative nature of the study limits the generalizability of the findings. Therefore, additional quantitative studies are necessary to isolate the effect of factors such as socioeconomic status, caregiver burden, and distance from treatment facilities on care-seeking.

Despite these limitations, the study has many strengths. One is that three different participant groups were interviewed, allowing for triangulation of the findings and a multifaceted view of the topic. In addition, caregivers were not recruited from a hospital setting; thus participants included caregivers who had not extensively sought medical care. The conceptual framework we developed provides a foundation for identifying and testing specific interventions. Furthermore, this study is the first to examine care-seeking behaviors for pediatric cerebral palsy in Ghana and can serve as a catalyst for future research on this topic. To ensure trustworthiness of the findings, the research results underwent peer scrutiny by members of the study team who were not directly involved in data collection, and their critiques were used to strengthen the analysis and interpretation of the data. Participants did not provide feedback on the findings of this study. However, the involvement of all the interviewers in the analysis and regular debriefing sessions during data collection ensured that the results reflected a diversity of participant experiences.

### Recommendations

One primary recommendation is for the Ghanaian government to include coverage of rehabilitation services for children, such as physiotherapy and occupational therapy, as part of the basic services for maternal and child health under the National Health Insurance Scheme (NHIS). This initiative would diminish the egregious impact of poverty on care-seeking and access to medical care as shown by our study. Evidence from Ghana NHIS demonstrates that abolishing user fees improved maternal and child health care utilization outcomes [38–40].

Policymakers should also support home-based management of cerebral palsy with continuous community-based rehabilitation training for caregivers by qualified providers. Home-based interventions eliminate the need for transportation, reduce caregiver burden, and preserve resources that health facilities or centers can use otherwise. In this study, the majority of caregivers transitioned from medical and alternative care to home-based management because they could easily manage that care, and there was no financial barrier. Home-based interventions for occupational therapy and physical therapy for pediatric cerebral palsy have had significant results [13]. A systematic review demonstrates that parenting interventions for caregivers with children with cerebral palsy have also had significant results in child behavioral outcomes [27].

Peer health navigation (PHN) interventions could improve home-based management. Currently, many caregivers seek support for home-based care from unofficial peer support groups. A PHN intervention would formalize and build on this existing support structure by training members of the caregiver community to act as peer health navigators [41]. Trained caregivers of children living with CP can support other mothers to navigate treatment expectations, identify and use the positive aspects of medical and alternative care, and provide lay counseling for couples to minimize divorce. They can also refer patients to services and empower their peers to break down their specific barriers to medical, alternative, and home-based care. Researchers have used PHNs successfully across diverse populations and multiple health conditions, such as HIV and cancer, to improve access to care and overall quality of life [41–43]. They are yet to be adapted for children living with a disability [44].

## Conclusion

Caregivers of children with cerebral palsy use alternative and home-based care to overcome barriers to medical care, address unmet treatment needs, and find solutions to specific cerebral palsy symptoms. Despite caregivers’ demonstrated desire and agency in taking charge of their children’s health, significant barriers exist in terms of negative views of disability, inadequate healthcare coverage, and poverty, forces that disempower some caregivers from using any form of care. Peer-based interventions, home-based care, and improved health insurance can dismantle barriers that prevent children with CP from living to their highest physical ability. Future studies should explore the effectiveness of home-based management of CP, particularly in low-resource settings.

## Supporting information

S1 Table Interview guide for health workers and complementary/alternative providers. (PDF)

S2 Table Interview guide for mothers of children with cerebral palsy. (PDF)

S1 File Case study: Transitioning between medical, alternative, and home-based care. (DOCX)
