# Supplementary material for: The multifaceted care-seeking practices among caregivers of children with cerebral palsy: Perspectives from mothers and providers in Ghana
Source: PLoS One. 2021 Oct 27;16(10):e0258650. doi: 10.1371/journal.pone.0258650 (PMC8550440; doi:10.1371/journal.pone.0258650)
Supplement: S1 Table — (PDF) [file pone.0258650.s001.pdf]

INTERVIEW GUIDE FOR HEALTH WORKERS AND COMPLEMENTARY/ALTERNATIVE PROVIDERS

LIFESTYLE AND HEALTH MANAGEMENT OF CHILDREN WITH CEREBRAL PALSY
What kind of care would you provide to a child with cerebral palsy? What else do you think a child with cerebral palsy might need?

1. **How often do you see children with cerebral palsy in your line ofwork?**
2. Please describe caregiver's adherence to the various treatment methods you provide/prescribe
3. **What is your opinion on the effectiveness of these treatments?**
4. In your opinion, how can mothers best manage the symptoms of their child at home?
5. In your opinion, how can spouses, community members, and family best support a child with cerebral palsy?
6. What does the ideal treatment regimen for children with cerebral palsy look like?
7. **What barriers do you think prevent you from being able to implement the ideal treatment regimen?**
8. What are the most important needs a child with cerebral palsy has?
9. How might you advise a mother in addressing non-medical needs of children with cerebral palsy?

SOCIAL PERCEPTIONS OF CEREBRAL PALSY
How do you think social perceptions of cerebral palsy impact the types of care that caregivers seek for them?

1. How do you think the general population perceives children with cerebral palsy?
2. How do you personally perceive children with cerebral palsy?
3. How do you think social perceptions of cerebral palsy affect how they are cared for?

OUTCOME EXPECTATIONS SURROUNDING DIFFERENT FORMS OF CARE FOR DEVELOPMENTAL DISABILITIES
What advice would you give to a new mother about seeking care for her child with cerebral palsy?

1. What do you think is challenging about seeking care within ahospital/health facility for cerebral palsy? What is good?
2. What do you think is challenging about seeking care outside of a hospital/health facility for cerebral palsy? What is good?
3. How do you think the health of a child with cerebral palsy will be affected if they are treated by a physiotherapist? A pediatrician? A herbalist? A religious leader? Any other provider that you can think of?
4. How do you work with other providers to provide care for children with cerebral palsy?
5. Which kind of provider do you think is the most important for a child withcerebral palsy?
6. **What is your opinion on the quality of care in the medical system?**
7. **What is your opinion on the quality of care outside of hospitals/health facilities?**
8. **Why do you think mothers seek care outside of hospitals/healthfacilities?**
9. **Why do you think mothers seek care within hospitals/health facilities?**

CHALLENGES WITH SEEKING CARE WITHIN HEALTH FACILITIES
How do you think health facilities can be changed to better cater to the needs of children with cerebral palsy?

1. **In your opinion, how often do mothers seek care within health facilities for children with cerebral palsy?**
2. **In your opinion, how often do mothers seek care outside of health facilities for**

**children with cerebral palsy?**
KNOWLEDGE ABOUT AVAILABLE MEDICAL/NON-MEDICAL RESOURCES FOR CHIDLREN WITH CEREBRAL PALSY<br>*What is your opinion on the effectiveness of medical/non-medical treatments for children with cerebral palsy?*
1. List any of the medical treatments you are aware of for cerebralpalsy<br>2. What is your opinion on the effectiveness of these treatments?<br>3. List any of the treatments you are aware of for cerebral palsy which take placeoutside of a hospital or health facility<br>4. What is your opinion on the effectiveness of these treatments?

***Who do you know in Accra that provides alternative care/care outside of the hospital for children with cerebral palsy?**
