# Supplementary material for: The multifaceted care-seeking practices among caregivers of children with cerebral palsy: Perspectives from mothers and providers in Ghana
Source: PLoS One. 2021 Oct 27;16(10):e0258650. doi: 10.1371/journal.pone.0258650 (PMC8550440; doi:10.1371/journal.pone.0258650)
Supplement: S2 Table — (PDF) [file pone.0258650.s002.pdf]

INTERVIEW GUIDE FOR MOTHERS OF CHILDREN WITH CEREBRAL PALSY

LIFESTYLE AND HEALTH MANAGEMENT OF CHILDREN WITH CEREBRAL PALSY

Describe the major strategies your use to manage your child's symptoms?

1. Please describe your experience raising a child with cerebral palsy
2. Describe how you manage the following common problems associated with cerebral palsy: feeding/swallowing difficulties, difficulties with toileting, difficulties with movement/stiffness, learning, epilepsy/convulsions, communication, behavioral issues
3. Walk us through a regular day with your child. What do you do? Where do yougo?
4. **What are the major challenges of caring for your child?**
5. What kind of support do you receive from your spouse, other family members, and your community in caring for your child?
6. Describe the major strategies your use to manage your child's symptoms at home. At the hospital?
7. What is your opinion on the effectiveness of these variousstrategies?
8. Which treatments do you use to care for your child that are not prescribed by a doctor or health worker?
9. **If you could have anything to treat your child, what would that be?**
10. What barriers do you think prevent you from being able to treat your child the way you want?
11. List all of the needs of your child. Which are most important?
12. **How do you address your child's social needs? (How do you support your child in interacting with other people?) Spiritual needs? (How do you support your child in their relationship with God?) Emotional needs? (How can you tell when your child is sad, happy, etc. and what do you do about it?)**

SOCIAL PERCEPTIONS OF CEREBRAL PALSY

What do you think people think about cerebral palsy? How does this affect how you care for your child?

1. How do you think others in your community perceive children withcerebral palsy? Your family? Your spouse?
2. Describe how you manage issues arising from stigma (shame, discrimination), stress, and marital stress as a result of your child's condition.
3. **How do you personally perceive children with cerebral palsy?**
4. How do you think social perceptions of cerebral palsy impact the types of care thatyou seek for your child?

OUTCOME EXPECTATIONS SURROUNDING DIFFERENT FORMS OF CARE FOR DEVELOPMENTAL DISABILITIES

Who do you feel most confident seeking care from? Why?

1. Describe your experience seeking care in the medical system for your child? What has been challenging? What has been good?
2. Describe your experience seeking care outside of hospitals/health facilities foryour child? What has been challenging? What has been good?
3. **How do you think the health of your child will be affected if they are treated by a physiotherapist? A pediatrician? A herbalist? A religious leader? Any other provider that you seek care from?**
4. What is your opinion on medical providers? Care providers who work outside of a hospital/health facility?
5. **What advice would you give to a new mother about seeking care for her child with cerebral palsy?**
6. What is your opinion on the quality of care in the medical system?
7. What is your opinion on the quality of care outside of hospitals/health facilities?

**8. Why do you seek care outside of hospitals/health facilities?**
CHALLENGES WITH SEEKING CARE WITHIN HEALTH FACILITIES<br>*What kinds of challenges do you face in seeking care from a health facility/hospital?*
1. What kinds of challenges do you face in seeking care from a physiotherapist? Pediatrician? Social worker? Nurse?<br>2. How often do you seek care within a health facility?<br>**3. How do you think health facilities can be improved to better cater to the needs of your child?**<br>4. Who do you feel most comfortable seeking care from within the medical system? Why?<br>5. Why or why not do you seek care within the medical system?
KNOWLEDGE ABOUT AVAILABLE MEDICAL/NON-MEDICAL RESOURCES FOR CHIDLREN WITH CEREBRAL PALSY<br>*What treatments are you most likely to seek for your child? Why?*
**1. What is your opinion on the effectiveness of these treatments? (medical/non-medical, refer to free list)**<br>**2. Do you intend to seek out any of these treatments? (medical/non-medical, refer to free list)**

***Who do you know in Accra that provides alternative care/care outside of the hospital for children with cerebral palsy?**
