# Supplementary material for: The multifaceted care-seeking practices among caregivers of children with cerebral palsy: Perspectives from mothers and providers in Ghana
Source: PLoS One. 2021 Oct 27;16(10):e0258650. doi: 10.1371/journal.pone.0258650 (PMC8550440; doi:10.1371/journal.pone.0258650)
Supplement: S1 File — (DOCX) [file pone.0258650.s003.docx]

# S1 File. Case study: transitioning between medical, alternative, and home-based care.

The experience of one study participant is summarized below as a cohesive example of a caregiver transitioning between multiple types of CP care. (The participant’s name has been changed for confidentiality.)

Joyce first heard the term “cerebral palsy” when she brought her 8 month-old son to the pediatric hospital for a fever. When her child received a CP diagnosis, Joyce didn’t understand what the condition meant or why the doctor recommended physiotherapy:

“Well, I just kept quiet. I thought it was just like malaria — you treat it and then it’s gone.”

After attending physiotherapy for several weeks, Joyce saw older children with CP and became curious about how long the condition would last. She decided to research cerebral palsy on her own and was devastated by what she found:

“I Googled it [cerebral palsy]. And that day I didn’t sleep. I wept throughout the night. Because as I was Googling it, I was just looking for a cure. And everywhere they would tell you there’s no cure. You can only manage it. So that day I wept.”

Desperate to find a solution for her son’s CP, Joyce began exploring alternative treatments:

“I started looking for answers. That is how I started roaming. I started going to places, pastors. I don’t know any pastor that has come on TV that I’ve never been to.”

Joyce believed that a few of the herbal treatments she tried were effective. However, she was dissatisfied that the wet herbal wraps left her child cold:

“[T]he child can even develop pneumonia because at times it is very cold and then the child has to be [wet]… I believe in herbal medicine. I know when you get the good ones [herbalists], they are very good, but right now, how to get the good ones is the problem.”

As a result, she transitioned back to medical care and consistently utilized physiotherapy. After about three years, Joyce decided to transition primarily to home-based care, citing finances, convenience, and self-efficacy as motivating factors:

“Yeah, I do it at home because I realized it [physiotherapy] got a bit expensive. And also when I go, they’d make me do everything by myself… So I don’t see why I should take a car all the way to Korle Bu [name of tertiary hospital]... Then all of a sudden they increased [the price] from 6 cedis to 35 cedis… So I realized it was like I was wasting my money going there and then doing it myself. So I just stopped.”

Joyce now performs at-home physiotherapy, supporting her son physically as he sits and walks. She supplements at-home treatment with prescription medication, often requesting specific prescriptions from physicians based on Internet research and advice from other mothers. Occasionally when she can afford the fee, Joyce will also take her child to speech therapy.
